# Extensive proteomic screening identifies the obesity-related NYGGF4 protein as a novel LRP1-interactor, showing reduced expression in early Alzheimer's disease

**DOI:** 10.1186/1750-1326-5-1

**Published:** 2010-01-14

**Authors:** Yuji Kajiwara, Sonia Franciosi, Nagahide Takahashi, Lisa Krug, James Schmeidler, Kevin Taddei, Vahram Haroutunian, Ulrik Fried, Michelle Ehrlich, Ralph N Martins, Samuel Gandy, Joseph D Buxbaum

**Affiliations:** 1 Laboratory of Molecular Neuropsychiatry, Mount Sinai School of Medicine, One Gustave L Levy Place, New York, NY 10029, USA; 2 Department of Psychiatry, Mount Sinai School of Medicine, One Gustave L Levy Place, New York, NY 10029, USA; 3 Department of Neuroscience, Mount Sinai School of Medicine, One Gustave L Levy Place, New York, NY 10029, USA; 4 Centre of Excellence for Alzheimer's Disease Research & Care, School of Exercise Biomedical and Health Sciences, Edith Cowan University, Joondalup, Western Australia, Australia; 5 Sir James McCusker Alzheimer's Disease Research Unit (Hollywood Private Hospital), Perth, Western Australia, Australia; 6 James J Peters Veterans Affairs Medical Center, 130 West Kingsbridge Road, Bronx, NY 10468, USA; 7 Department of Neurology, Mount Sinai School of Medicine, One Gustave L Levy Place, New York, NY 10029, USA; 8 Department of Genetics and Genomic Sciences, Mount Sinai School of Medicine, One Gustave L Levy Place, New York, NY 10029, USA

## Abstract

**Background:**

The low-density lipoprotein receptor related protein 1 (LRP1) has been implicated in Alzheimer's disease (AD) but its signalling has not been fully evaluated. There is good evidence that the cytoplasmic domain of LRP1 is involved in protein-protein interactions, important in the cell biology of LRP1.

**Results:**

We carried out three yeast two-hybrid screens to identify proteins that interact with the cytoplasmic domain of LRP1. The screens included both conventional screens as well as a novel, split-ubiquitin-based screen in which an LRP1 construct was expressed and screened as a transmembrane protein. The split-ubiquitin screen was validated in a screen using full-length amyloid protein precursor (APP), which successfully identified FE65 and FE65L2, as well as novel interactors (Rab3a, Napg, and ubiquitin b). Using both a conventional screen as well as the split-ubiquitin screen, we identified NYGGF4 as a novel LRP1 interactor. The interaction between LRP1 and NYGGF4 was validated using two-hybrid assays, coprecipitation and colocalization in mammalian cells. Mutation analysis demonstrated a specific interaction of NYGGF4 with an NPXY motif that required an intact tyrosine residue. Interestingly, while we confirmed that other LRP1 interactors we identified, including JIP1B and EB-1, were also able to bind to APP, NYGGF4 was unique in that it showed specific binding with LRP1. Expression of NYGGF4 decreased significantly in patients with AD as compared to age-matched controls, and showed decreasing expression with AD disease progression. Examination of Nyggf4 expression in mice with different alleles of the human *APOE4 *gene showed significant differences in Nyggf4 expression.

**Conclusions:**

These results implicate NYGGF4 as a novel and specific interactor of LRP1. Decreased expression of LRP1 and NYGGF4 over disease, evident with the presence of even moderate numbers of neuritic plaques, suggests that LRP1-NYGGF4 is a system altered early in disease. Genetic and functional studies have implicated both LRP1 and NYGGF4 in obesity and cardiovascular disease and the physical association of these proteins may reflect a common mechanism. This is particularly interesting in light of the dual role of ApoE in both cardiovascular risk and AD. The results support further studies on the functional relationship between NYGGF4 and LRP1.

## Background

The low-density lipoprotein (LDL) receptor-related protein 1 (LRP1) is a multifunctional receptor that mediates the internalization and degradation of ligands involved in diverse metabolic pathways [1]. LRP1, which is expressed by many cells in the central nervous system, including neurons and astrocytes [2,3], is synthesized as a 600-kDa polypeptide that is subsequently cleaved by furin in the trans-Golgi compartment into two subunits of 515 and 85 kDa [4,5]. The 515-kDa subunit contains the ligand binding domains and remains noncovalently associated with the 85-kDa subunit, which includes the transmembrane domain and a short cytoplasmic tail. LRP1 plays an important role in several physiological processes including embryonic development [6].

LRP1 recognizes and internalizes numerous extracellular ligands, including protease/protease inhibitor complexes and apolipoprotein particles such as apolipoprotein E (ApoE), and has an important role in intracellular signaling pathways, some being mediated through tyrosine phosphorylation sites in the cytoplasmic domain [1,7-9]. The binding of the AD-associated apolipoprotein ApoE to LRP1 has been shown to be neuroprotective [10]. LRP1 also interacts with other AD-related proteins including APP, BACE1 and presenilin 1 [11-13]. The cytoplasmic tail of LRP1 contains two NPXY motifs and two dileucine-based motifs and may interact with multiple adaptor and scaffolding proteins, include PSD-95, Shc, Mint2, disabled-1 (Dab1), JIP-1, JIP-2 and Fe65 [14-16]. Signaling pathways associated with LRP1 include intracellular cyclic adenosine monophosphate (cAMP), protein kinase A (PKA), calcium signaling via N-methyl-D-aspartate (NMDA) receptors [17] and mitogen-activated protein (MAP) kinase pathway [18].

LRP1 has been shown to be associated with late onset AD in some studies but not in others [19-21]. Age of onset and severity of AD has also been shown to be associated with LRP1 [19,22]. In addition, LRP1 signaling pathways have been functionally implicated in AD since LRP1 has been found to be associated with senile plaques in AD brains along with the LRP1 ligands ApoE, α-2 macroglobulin and APP, which themselves are genetically and functionally associated with AD [23-25]. LRP1 has been implicated as a receptor for cellular uptake of Aβ [22,26,27] as well as in the efflux of Aβ at the blood brain barrier [28]. Furthermore, LRP1 has also been shown to promote the generation of Aβ through an interaction of the Kunitz protease inhibitor domain (KPI) and cytoplasmic tail with APP [29,30]. The cytoplasmic tail alone has also been shown to promote delivery of APP to lipid raft microdomains that are enriched with BACE1 activity [31]. These results indicate that LRP1 plays a role in Aβ generation, uptake into cells and removal from the CNS.

LRP1 contains four putative ligand binding domains (I, II, III and IV). The furin endopeptidase processing site is found between the fourth ligand binding domain and the transmembrane region. The cytoplasmic tail of LRP1 contains at least five motifs: two NPXY motifs, two dileucine motifs, and one YXXL motif, each of which can have a role in LRP1 trafficking and signaling. The YXXL motif has been shown to serve as the dominant endocytosis signal for LRP1 [32], whereas the two NPXY motifs have been shown to be subject to tyrosine phosphorylation and constitute binding sites for proteins with phosphotyrosine binding (PTB) domains. One of the NPXY motifs interacts with the transcription factor Fe65 [14].

In this study, we used conventional yeast two-hybrid screens and a novel split-ubiquitin yeast two-hybrid screen to identify novel interactors of LRP1. NYGGF4 was found to be a novel, and specific, interactor of LRP1, whose expression is regulated by ApoE allele in a mouse model is decreased during AD progression. These results further implicate LRP1 as well as of the novel LRP1 interactor, NYGGF4, in AD pathogenesis as well as obesity and cardiovascular disease.

## Results

### Yeast two-hybrid analysis with cytoplasmic domains of LRP1

**Figure 1:**
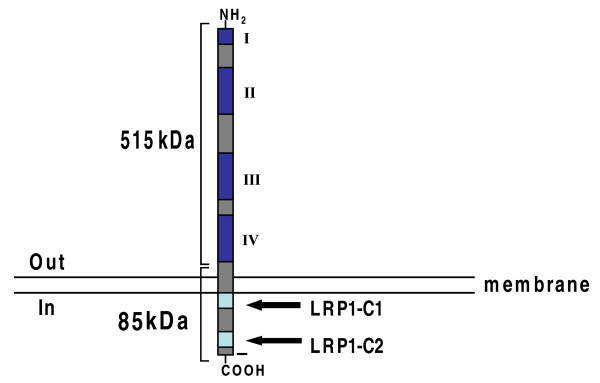
**Schematic of LRP1**. The 515-kDa extracellular region of LRP1 consists of four domains: I, II, III and IV. The 85-kDa intracellular region of LRP1 includes two NPXY motifs.

Baits derived from two different cytoplasmic domain sequences of LRP1 (Fig. 1), containing either the first or second NPXY motif, were used to screen for interacting proteins in a mouse brain library. One bait (LRP1-C1) consisted of amino acids 4455-4476 of the LRP1 cytoplasmic domain and the other of amino acids 4497-4533 (LRP1-C2). After validating that the fusion proteins were expressed in yeast and that each by itself did not activate reporter gene expression, 1.1 × 10^7 ^colonies (for LRP1-C1) and 2.8 × 10^7 ^colonies (for LRP1-C2) from an adult mouse brain cDNA library (complexity 1 × 10^6^; Clontech) were screened. Clones identified in the screen were re-assayed with an X-Gal filter test and those passing this test were further analyzed by retransformation with either the LRP1 bait or with a control bait. Restriction analysis and sequencing of all specific clones was then carried out (Table 1). The results for LRP1-C2 include previously identified and validated LRP1 interactors (EB-1, JIP1b) as well as novel interactors (Nyggf4, Hnrpdl). The overlap with prior results for LRP1-C2 confirm the approach, while results for LRP1-C1 represent the first successful published screen with this domain and identified Ric-8b, Freud-1 and the ribonucleoprotein U1 as interactors.

**Table 1:** Results of traditional yeast two-hybrid screens

Prey	Number of clones	Accession number
**Using LRP1-C1 as bait**		

Ric-8b	3	NM_001013441

Freud-1	1	NM_145970

Small nuclear ribonucleoprotein U1	1	NM_009224

**Using LRP1-C2 as bait**		

JIP1b	22	NM_011162

EB-1	3	XM_001003394

Nyggf4	3	NM_001003948.1

Hnrpdl	1	NM_016690

### Split-ubiquitin yeast two-hybrid analyses

A novel two-hybrid method has been developed to allow screening transmembrane proteins for interactors [33]. We first carried out this split-ubiquitin, yeast two-hybrid screen for full-length APP_695 _to determine whether this approach would identify previously validated, as well as possibly novel, APP interactors. Because of the nature of the screen (transmembrane bait but soluble prey), we focused on soluble proteins and identified five specific, unique clones (Table 2), including Fe65 and Fe65L2, as well as rab3a, Napg, and ubiquitin b. The identification of Fe65 proteins in this screen validated the approach and justified its use with LRP1.

**Table 2:** Results of the split-ubiquitin yeast two-hybrid screens

Prey	Number of clones	Accession number
**Using APP_695 _as bait**		

Fe65	1	NM_009685

Fe65l2	1	NM_146085

Rab3a	1	NM_009001

Napg	1	NM_028017.1

Ubiquitin b	1	NM_011664.3

**Using mLRP4 as bait**		

Nyggf4	2	NM_001003948.1

Screening the same mouse brain library with the LRP1 transmembrane mini-receptor mLRP4, we identified 1 unique soluble clone, Nyggf4 (Table 2). The specificity of the interaction was validated, showing no interaction in a follow up screen with irrelevant baits (p53 and lamin A/C). It was exciting to note that Nyggf4 was identical to the protein we previously identified with conventional yeast two-hybrid screen using the LRP1-C2 bait (Table 1). The identification of Nyggf4 in two very different yeast two-hybrid assays provided evidence that this protein may be an important LRP1-interacting protein.

### Validation of the LRP1-Nyggf4 interaction

**Figure 2:**
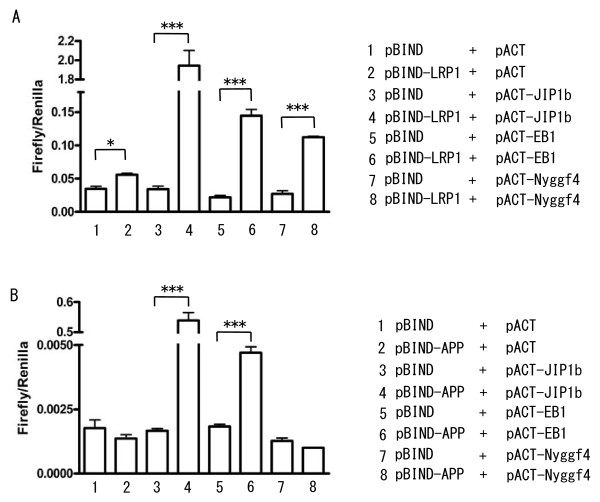
**Mammalian two-hybrid assays with the LRP1 or APP cytoplasmic domains**. H4 human neuroglioma cells were transfected with the indicated constructs, and luciferase activity measured. **A**. The LRP1 cytoplasmic domain was cloned into the pBIND vector while the interactors JIP-1b, EB-1 and Nyggf4 were cloned into the pACT vector. Strong interactions between LRP1 and JIP-1b, EB-1 or Nyggf4 were observed, confirming results with yeast two hybrid studies. **B**. The APP cytoplasmic domain (known as APP intracellular domain/AICD) was cloned into pBIND, while the interactors JIP-1b, EB-1 and Nyggf4 were cloned into pACT. Strong interactions between JIP-1b or EB-1 with the APP intracellular domain were observed but no interaction was observed with Nyggf4 and the APP intracellular domain. ***, P < 0.0001.

The interaction between LRP1 and Nyggf4 was confirmed by four additional methods. We first used a mammalian two-hybrid system to confirm that LRP1 and Nyggf4 interact in mammalian cells (Fig. 2A). Nyggf4 showed significant interaction with the LRP1 cytoplasmic domain in this assay. For comparison, we included additional potential LRP1 interactors, identified in the screen with the LRP1-C2 bait. From these latter studies, we confirmed that both EB-1 and JIP1b were LRP1 interactors in this system (Fig. 2A).

It was of interest to us that some of the LRP1-C2 domain interacting proteins identified by us and by others (e.g., JIP1b and EB-1, [34,35]) have also been reported to interact with APP. We therefore sought to determine whether Nyggf4 could interact with APP (Fig. 2B). Using the mammalian two-hybrid system, we observed that under conditions where JIP1b and EB-1 could bind the cytoplasmic domain of APP, Nyggf4 could not. The Nyggf4 interaction shows specificity to LRP1 in these studies, while JIP1b and EB-1 do not, as they bind both LRP1 and APP.

**Figure 3:**
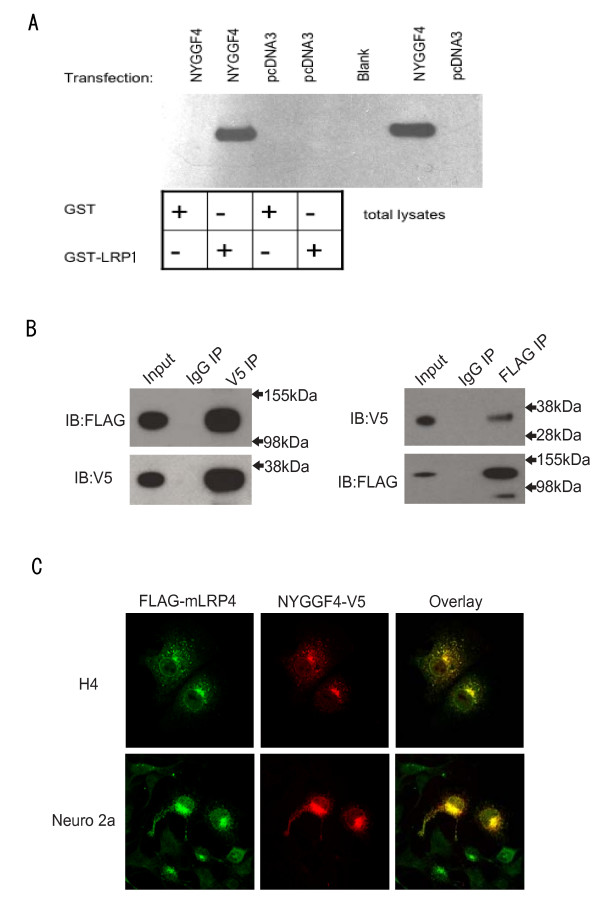
**Interaction of LRP1 and NYGGF4 expressed in mammalian cells**. **A**. COS-7 cells were transfected with vector (pcDNA3) or NYGGF4-V5 cloned into pcDNA3.1V5/His and resultant cell lysates were subjected to GST-pulldown with either GST or with GST-LRP1 (cytoplasmic domain only), as indicated by the scheme at the base of the figure. Aliquots of cell lysates (right most lanes) and aliquots of the GST-pulldowns were probed by immunoblotting with anti-V5 antibody for NYGGF4. NYGGF4 is precipitated with GST-LRP1, but not with GST alone. **B**. FLAG-tagged mLRP4 and V5-tagged NYGGF4 were cotransfected into H4 neuroglioma cells, and immunoprecipitation (IP) of either protein by FLAG or V5 antibody successfully coprecipitated the other protein as detected by immunoblot (IB). Lysate was also blotted to show expression in transfected cells (Input) and control immunoprecipitations were carried out with purified control immunoglobulin (IgG). **C**. FLAG-tagged mLRP4 and V5-tagged NYGGF4 were cotransfected into either H4 (upper) or Neuro 2A (lower) cells, and detected by immunocytochemistry. Both proteins showed extensive colocalization, especially in the perinuclear region. Results are representative of three independent experiments.

We also confirmed an interaction between LRP1 and NYGGF4 using GST pulldown, coimmunoprecipitation and colocalization studies. For the GST pulldowns, a fusion protein comprised of GST and the LRP1-intracellular domain (GST-LICD), but not GST alone, could precipitate NYGGF4, when it was expressed in COS-7 cells (Fig. 3A).

Subsequently, FLAG-mLRP4 and NYGGF4-V5 were cotransfected into H4 neuroglioma cells, and immunoprecipitation of either protein by FLAG or V5 antibody successfully coprecipitated the other protein as detected by immunoblot (Fig. 3B). Furthermore, both proteins showed extensive colocalization, especially in the perinuclear region, when cotransfected into either H4 cells or Neuro 2A cells (Fig. 3C).

### Characterization of the LRP1-Nyggf4 interaction

**Figure 4:**
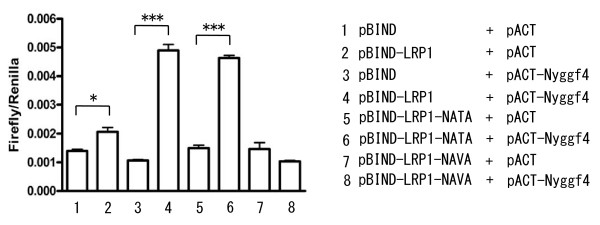
**Mammalian two-hybrid assays with wild-type and mutant LRP1**. NPXY motifs of the cytoplasmic domain of LRP1 were mutated and the interaction of the cytoplasmic domain with Nyggf4 was assessed as described in Fig. 2. NATA represents the mutation of the NPXY motif of LRP1-C1 and NAVA represents the mutation of NPXY motif of LRP1-C2. Nyggf4 binds to wild-type LRP1 and to LRP1-NATA but not to LRP1-NAVA. *, P < 0.01; ***, P < 0.001.

We next used the mammalian two-hybrid system to determine whether the LRP1-Nyggf4 interaction required the intact NPVY motif of the LRP1-C2 domain (Fig. 4). Nyggf4 interacted with wild-type LRP1 intracellular domain (LICD), but not with LICD where the NPVY motif in C2 domain was mutated to NAVA. In contrast, mutating the NPTY motif in C1 domain to NATA in the LRP1 domain had no effect on Nyggf4 binding. This indicated both that the binding of Nyggf4 involved a canonical NPXY motif and was specific for the motif in LRP1-C2.

### Expression of NYGGF4 mRNA in Alzheimer's disease

**Figure 5:**
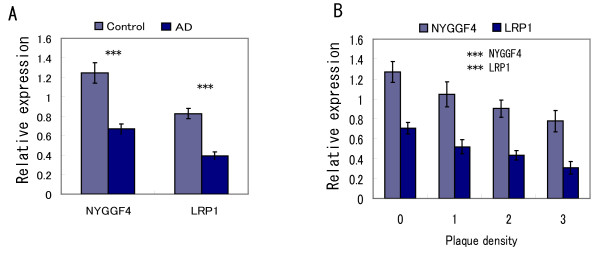
**Expresssion of LRP1 and NYGGF4 in Alzheimer disease**. **A**. Expression of LRP1 and NYGGF4 in entorhinal cortex (BA28/36), comparing controls and subjects with AD. ***, p < 0.001 **B**. Expression of LRP1 and NYGGF4 as a function of neuritic plaque density. Plaque density scores are derived from counts across five cortical regions: 0, no neuritic plaques; 1, 1-6 plaques per mm^2^; 2, 7-12 plaques per mm^2^; and, 3, >12 neuritic plaques per mm^2^. ***, p < 0.001 for negative linear trend.

Given the potential role for NYGGF4 as an LRP1-interacting protein and a role for LRP1 in AD, we then asked whether there was altered expression of mRNA for Nyggf4 and LRP1 in that disease (Fig. 5A). Expression of human NYGGF4 was significantly reduced in probable and definite AD as compared to age-matched controls (F_1,88 _= 15.419, p < 0.001). Interestingly, expression of NYGGF4 decreased over the progression of the disease, as assessed by either CDR scores (F_6,90 _= 3.917, p < 0.01; data not shown), Braak stage (F_5,82 _= 8.979, p < 0.001; data not shown), or increasing plaque density (F_3,94 _= 4.096, p < 0.01; Fig. 5B). The levels of NYGGF4 decreased with even the smallest increase in plaque densities. The associations between NYGGF4 levels and disease progression all demonstrated highly significant linear trends (F_1,95 _= 17.425, p < 0.01 for CDR score, F_1,85 _= 25.053, p < 0.001 for Braak stage, and F_1,96 _= 13.102, p < 0.001 for plaque density).

LRP1 was also significantly reduced (39% reduction) in probable and definite AD, as compared to age-matched controls (F_1,88 _= 15.877, p < 0.001; Fig. 5A). LRP1 expression also showed decreased expression over the course of disease, as measured by CDR score, Braak stage, or plaque load (Fig. 5B, and data not shown). The decrease in LRP1 with increasing Braak stage was significant (F_5,82 _= 5.462, P < 0.001) and the linear trend was significant as well (F_1,86 _= 9.381, p < 0.01). Although the decrease of LRP1 with increasing CDR score was not significant (F_6,90 _= 1.65, p = 0.143), the linear trend was significant (F_1,95 _= 4.832, p < 0.05). Finally, LRP1 expression was significantly reduced (30% for group 1, 37% for group 2, and 54% for group 3, all compared to group 0) with increasing neuritic plaque load (F_3,94 _= 7.650, p < 0.001; Fig. 5B) with a strong negative linear trend (F_1,96 _= 22.215, p < 0.001).

### Expression of Nyggf4 mRNA in mouse models for Alzheimer's disease

**Figure 6:**
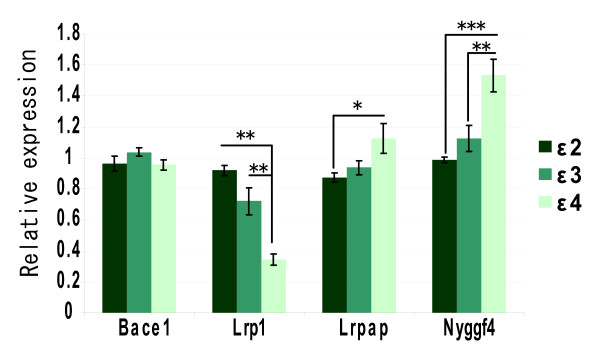
**Expression of Lrp1, Nyggf4, Bace1, and Lrpap in a mouse model of Alzheimer's disease**. Relative mRNA expression levels of *Bace1*, *Lrp1*, *Lrpap*, and *Nyggf4 *from whole brain tissue of mice expressing either the ApoE2, ApoE3, or ApoE4 isoform. All values are normalized to three ubiquitously expressed housekeeping genes (*Actb*, *Gapdh*, and *Rpl13a *using qBase. *, p < 0.05, **, p < 0.01, and ***, p < 0.001.

Subsequently, we asked whether there was altered expression of mRNA for Nyggf4 and Lrp1 in mice with different human APOE isoforms, as LRP1 is a major receptor for APOE and APOE is a major AD risk factor (Fig. 6). Interestingly, expression of Nyggf4 was significantly upregulated in mice carrying human APOE ε4 gene compared to mice carrying human APOE ε2 and human APOE ε3 gene (F_1, 17 _= 5.992, p < 0.001 and F_1,17_, = 0.008, p < 0.01, respectively). Furthermore, expression of Lrp1 was significantly decreased in these mice (F_1, 17 _= 0.312, p < 0.001 for APOE ε2 and F_1,17_, = 16.463, p < 0.01, for APOE ε3 respectively). For comparison, Bace1 levels were unchanged in the mice while another Lrp1-binding protein, Lrpap showed increased expression in the ApoE4 mice, similar to Nyggf4 (F_1, 17 _= 2.695, p < 0.05 for APOE ε2 and F_1,17_, = 1.012, p = 0.079, for APOE ε3 respectively).

## Discussion

In the current study, we screened for novel interactors of the LRP1 intracellular domain using both conventional and split-ubiquitin screens (see Fig. 1). Two separate baits, each encompassing one of the two NPXY motifs of the LRP1 intracellular domain (Figure 1), were used in the conventional screens (Table 1). For the split-ubiquitin screen, mLRP4, which consisted of domain IV, transmembrane and cytoplasmic tail of LRP1 (Figure 1) was used, after validating the approach with full-length APP (Table 2). NYGGF4 was identified as an LRP1 interactor using both LRP1-C2 in the conventional screen as well as in the slit-ubiquitin screen (Tables 1 and 2). This interaction was validated as specific in yeast, as well as in mammalian two-hybrid screens and in colocalization and coprecipitation experiments. We observed that the interaction of NYGGF4 with LRP1 is mediated by the NPVY sequence in LRP1. Interestingly, unlike other LRP1-binding proteins, which can also interact with APP, NYGGF4 was shown to bind LRP1 but not APP, indicating that the NYGGF4-LRP1 interaction shows specificity not seen with some other interactors.

NYGGF4 is a 250 amino acid cytoplasmic protein that is largely defined by single PTB domain. Amongst the clones identified in this study, all contained the PTB domain with flanking sequence supporting a role for this PTB domain in the interaction with LRP1. This conclusion is supported by a recent study that used numerous PTB domains to screen for interacting proteins [36]. In that study, the PTB domain of NYGGF4 (there identified as Q7Z2X4), was shown to pull down a complex of LRP1 and cubulin, the latter previously associated with megalin, which is another member of the LDLR family.

Altogether, NYGGF4 remains a poorly characterized gene that was first identified as a gene showing increased expression in obese subjects [37]. Those studies demonstrated high expression of NYGGF4 in adipose tissue, heart and skeletal muscle and showed that NYGGF4 increased proliferation of 3T3-L1 preadipocytes. Later studies by the same group showed a role for NYGGF4 in glucose homeostasis in mature adipocytes, with increased expression of NYGGF4 leading to reduced insulin-stimulated glucose uptake and impaired insulin-stimulated GLUT4 translocation [38]. When fed with a high fat, high sucrose (diabetogenic) diet to induce obesity, ApoE deficient mice showed no differences in plasma lipid levels, lipoprotein profiles or atherosclerotic lesion areas [39] and are hence resistant to many of the effects of the diabetogenic diet. In a similar vein, disruption of LRP1 in adipocytes led to a delayed postprandial lipid clearance, reduced body weight, smaller fat stores, lipid-depleted brown adipocytes, and improved glucose tolerance, while showing resistance to dietary fat-induced obesity and glucose intolerance [40]. These results, when taken together with our studies, lead us to hypothesize that the LRP1-NYGGF4 interaction may mediate critical effects of LRP1 on diet-induced obesity, glucose tolerance, and cardiovascular risk factors. In support of this, review of SNPs in NYGGF4 analyzed in a large study [41] indicate that two (rs2215598 and rs6739369) show nominal association with hypertension (P = 0.00021 and P = 0.00008, respectively).

The very strong association of ApoE with AD also raises the question as to the role of ApoE receptors such as LRP1, and the molecules associated with them, in AD. It was interesting to observe that expression of NYGGF4 is decreased in AD (Fig. 5). Moreover the levels of NYGGF4, as well as LRP1, decreased as a function of disease progression, particularly as defined by increasing neuritic pathology. Further studies would need to address the mechanisms of altered expression of these genes in AD progression, which might include loss of neurons as well as additional mechanisms. Interestingly, the functional linkage between ApoE, LRP1, and NYGGF4 receives support from our observations in ApoE-transgenic mice, where ApoE genotype modulates expression of Lrp1 mRNA and expression of Nyggf4 mRNA in opposite directions. The mechanism for this would require further experimentation, however it appears that levels of this ApoE binding protein and its associated adaptor are regulated by ApoE isoforms in such a way that would likely lead to alterations in downstream signaling. How this might modulate cardiovascular risk or cognitive function is of course a very interesting question for future studies.

## Conclusions

Extensive proteomic analyses, including yeast-two hybrid screening, mammalian two-hybrid screening, copreciptation, and colocalization, identify NYGGF4 as an LRP1-binding protein. NYGGF4 is not well studied but appears well situated to mediate the effects of LRP1 on aspects of obesity, glucose tolerance, and cardiovascular risk factors. Moreover, the relationship between ApoE genotype and NYGGF4 expression supports an important relationship between these two proteins in AD risk. Further study of NYGGF4 in the CNS and the periphery will clarify the role of the protein in physiological processes and in disease.

## Methods

### Two-hybrid screening

All yeast two-hybrid screening was carried out together with Dualsystems Biotech (Zurich). For conventional yeast two-hybrid screening, baits flanking either the first (LRP1-C1, ^4445^KRRVQGAKGFQHQRMTNGAMNVEIG**NPTY**KMY^4476^) or second (LRP1-C2, ^4497^KPTNFT**NPVY**ATLYMGGHGSRHSLASTDEKRELLGR^4533^) NPXY motifs of LRP1 (shown above in bold lettering) were cloned into the DUALhybrid bait vector in frame with the LexA DNA-binding domain, and the resultant construct confirmed by sequencing. After validating that baits, by themselves, did not activate reporter gene expression, an adult mouse brain cDNA library was screened (Clontech, Mountain View, CA). Bait dependency of each putative clone was confirmed by retransforming into strains carrying either the relevant LRP1 construct or a control vector. All remaining baits were then sequenced.

Split-ubiquitin yeast two-hybrid screening, which has the advantage that it allows for screening with membrane-inserted transmembrane proteins, was carried out as previously described [33]. To first validate the method, we used full-length amyloid precursor protein (APP) as bait, onto which we fused (at the COOH-terminal) the COOH-terminal portion of ubiquitin ("Cub", see [33]) and an artifical transcription factor (LexA-VP16). A mouse brain cDNA library with a mutated version of the amino-terminal portion of ubiquitin ("NubG", see [33]), fused to the amino-terminal of prey sequences was used to screen for potential positive interactors. Specific clones remaining after false positives were eliminated by the use of irrelevant baits (p53 and lamin A/C) were sequenced.

Since LRP1 is a large protein not readily expressed in heterologous systems, minireceptors that include ligand-binding domains of LRP1 have been generated [42]. The minireceptor mLRP4 consists of the signal peptide, a FLAG tag, and the entire sequence from ligand binding domain IV through the COOH-terminal of the native receptor. As with APP, Cub and an artificial transcription factor (LexA-VP16) were fused to the COOH-terminal of mLRP4 (a gift of Dr. Guojun Bu) and screening was carried out as described above.

### GST pulldown experiments

GST pulldowns were carried out as previously described [43]. Briefly, COS-7 cells were transfected with control vector or with NYGGF4-V5 using Lipofectamin 2000 (Invitrogen), according to the manufacturer's instructions, and lysed after 24-48 hrs. Subsequently, purified GST or GST fused to the cytoplasmic domain of LRP1 (beginning at the KRR membrane anchor) previously incubated with glutathione beads were incubated with 100 μg of cell lysate. After washing three times with wash buffer, beads were boiled in SDS sample buffer and run on 10% SDS-PAGE gel. Proteins were analyzed by immunoblotting and ECL detection.

### Mammalian two-hybrid assays

To confirm interactions in mammalian cells, the Checkmate mammalian two-hybrid system (Promega) was used according to manufacturer's instructions. H4 neuroglioma cells were transfected with various combinations of vectors expressing the following proteins: a naked GAL4 DNA binding domain (pBIND); a GAL4 DNA binding domain fused to the intracellular domain of LRP1 (pBIND-LRP1; again beginning at the KRR membrane anchor) or APP (pBIND-APP); a naked VP16 transactivation moiety activation domain (pACT); a VP16 transactivation moiety fused to JIP1b (pACT-JIP1b), EB-1 (pACT-EB-1) or Nyggf4 (pACT-Nyggf4) (the lower case font for Nyggf4 here and elsewhere in the manuscript is to indicate that the murine sequence was used). A reporter construct with a GAL4 consensus binding sequence upstream of firefly luciferase coding sequence was also cotransfected in all conditions. Interactions were monitored by transactivation of luciferase expression. Both firefly luciferase (reflecting gene activation) and Renilla luciferase (reflecting transfection efficiency) were measured in the same sample at the same time, leading to much reduced noise. Data were obtained from three or more independent experiments and expressed as a ratio of firefly luciferase to Renilla luciferase expression (Firelfy/Renilla).

### Coimmunoprecipitation experiments

H4 neuroglioma cells were cotransfected with FLAG-mLRP4 and NYGGF4-V5 plasmids for 24 hours using Fugene6. Cells were lysed in lysis buffer containing 1% Triton X100 and protease inhibitor cocktail in 1× phosphate buffer saline (PBS). 100 μg of lysates were dissolved in 500 μl of lysis buffer and incubated with 5 μg of antibody or control IgG for 2 hours, then with additional Protein-G agarose beads for 1 hour at 4°C. Beads were washed with wash buffer containing 0.5% Triton X100 in PBS three times and then boiled in SDS loading buffer. Immunoblotting was performed as described in GST pulldown.

### Colocalization experiments

H4 or Neuro-2A cells were cotransfected with FLAG-mLRP4 and NYGGF4-V5 using Fugene6 (Roche) for 24 hours and immunostained with rabbit 1704 anti-LRP1 and mouse anti-V5 antibodies, then subsequently with AlexaFluor 488 anti-rabbit IgG and AlexaFluor 594 anti-mouse IgG (Invitrogen) antibodies. The 1704 anti-LRP1 antibody was kindly provided by Dr. Edward Koo.

### Gene expression in postmortem brains

All assessments and post-mortem procedures were approved by the Institutional Review Boards of Pilgrim Psychiatric Center, Mount Sinai School of Medicine, and the James J. Peters VA Medical Center. Subject selection, cognitive assessment and neuropathological assessment were carried out as previously described [44]. Briefly, frozen post-mortem brain tissue from the entorhinal cortex (Brodmann area 28/36) of subjects diagnosed with or without AD (normal neuropathology, N = 33; definite AD, N = 52; probable AD, N = 9; possible AD, N = 8) were obtained from the Mount Sinai/Bronx Veterans Administration (VA) Medical Center Department of Psychiatry Brain Bank. Normal controls had no history of any psychiatric or neurological disorders and no discernible neuropathological lesions. Neuritic plaque density was quantified in 5 cortical regions [Brodmann area 9 (BA9; middle frontal gyrus), Brodmann area 45/47 (BA45/47; orbital frontal gyrus), Brodmann area 21/22 (BA21/22; superior temporal gyrus), Brodmann area 39 (BA39; inferior parietal cortex), and Brodmann area 17 (BA17; calcarine cortex)], as previously described [45]. The results from these analyses were then used to bin the samples into gour groups: Samples with a score of 0, corresponded to those with no plaques observed in the 5 cortical regions; a score of 1 was used for samples with only 1-6 neuritic plaques per mm^2^; and a score of 2 and 3 were used for samples with 7-12 and >12 neuritic plaques per mm^2^, respectively.

Total RNA extraction and reverse transcriptase (RT) reactions were performed after DNAse treatment and template RNA quality, including degradation and DNA contamination, were controlled. Taqman^® ^probes for endogenous control gene (RPLP0, Hs99999902_m1; GUSB, Hs99999908_m1; B2M, Hs99999907_m1) and experimental probes (LRP1, Hs01059330_m1; NYGGF4, Hs00952182_m1) were purchased from Applied Biosystems Inc. and the reactions were carried out with TaqMan^® ^Universal PCR Master Mix (430437, Roche) using ABI Prism 7900HT at Mount Sinai Quantitative PCR Shared Research Facility. Expression levels of each sample were normalized to the geometric mean of GUSB, B2M, and RPLP0 expression levels.

### Gene expression in mouse models for Alzheimer's disease

Male APOE knock-in (KI) mice, homozygous for human APOE *2, *3 and *4 were originally obtained from Taconic (Germantown, NY, USA) and a colony maintained at the Animal Resources Centre (ARC, Perth, Western Australia). These mice have been previously described [46-50]. RNA was extracted from whole brain of male C57BL mice expressing either the apolipoprotein ε2 (ApoE2) (n = 10), ApoE3 (n = 10), or ApoE4 (n = 9) isoform using an RNeasy Mini-Prep Kit (Qiagen). The quality of all RNA samples was measured using an RNA 6000 Nano Kit (Agilent Technologies) and each sample used for subsequent cDNA synthesis possessed an RNA Integrity Number (RIN) of 0.9 or higher. cDNA was generated from total RNA extracts using a High Capacity cDNA Archive Kit (Applied Biosystems) as per manufacturer instruction. mRNA levels of *Bace1*, *Lrp1*, *Lrpap*, and *Nyggf4 *were determined via qPCR using the appropriate TaqMan UPL probe and primer sets (Applied Biosystems). Four ubiquitously expressed housekeeping genes (*Actb*, *Gapdh*, *18s*, and *Rpl13a*) were used as references and all data was normalized using qBase [51]. qBase selected three of the housekeeping genes (*Actb*, *Gapdh*, and *Rpl13a*) as endogenous references based on their geometric mean expression.

### Statistical analyses

Statistical analyses were performed using SPSS 11.0 software. For mammalian two- hybrid data, a t-test was used to compare effects of either LRP1 or APP on Firefly/Renilla ratio with each of the interactors or to vector alone. For expression data, an ANCOVA was carried out with LRP1 and NYGGF4 as dependent variables and either case status, CDR score, Braak Stage, or neurtic pathology scores as independent variables. Since data for NYGGF4 expression were not normally distributed, NYGGF4 data was log transformed. In each analysis, we used post-mortem interval, sex, sample pH, or ApoE4 allele as covariates if they were associated with the dependent variable. Sex showed no associations in LRP1 expression versus CDR score and Braak stage and was therefore omitted from the analysis. We also assessed the linear relationship by linear regression analysis controlling for covariates. All data are presented as means ± SEM and significant findings were noted when p < 0.05.

## Competing interests

The authors declare that they have no competing interests.

## Authors' contributions

YK, SF, NT, LK and UF carried out experiments in Figures 1, 2, 3, 4, 5, 6. ME, KT and RM provided mice for the experiments. JS provided statistical oversight. VH provided all postmortem samples and the all subgrouping of the samples by CDR, Braak, and plaque staging. YK, SF, NT, VH, ME, RM, SG, and JDB developed the manuscript. JDB directed all experimental studies. All authors read and approved the final manuscript.
